# YBX1 mediates autophagy by targeting p110β and decreasing the sensitivity to cisplatin in NSCLC

**DOI:** 10.1038/s41419-020-2555-4

**Published:** 2020-06-19

**Authors:** Yanwei Cui, Fengzhou Li, Qiang Xie, Shilei Zhao, Tao Guo, Ping Guo, Sheng Hu, Jiaojiao Hao, Chunfang Tian, Wendan Yu, Zhuoshi Li, Lei Fang, Lei Zhao, Manyu Chen, Taihua Wu, Chundong Gu

**Affiliations:** 1 Department of Thoracic Surgery, The First Affiliated Hospital of Dalian Medical University, 116011 Dalian, China; 2 Departments of Respiratory Medicine, Zhongshan Hospital, Dalian Univerdity, 116011 Dalian, China; 3 Institute of Cancer Stem Cell, Dalian Medical University, 116011 Dalian, China; 4 Departments of Respiratory Medicine, The First Affiliated Hospital of Dalian Medical University, 116011 Dalian, China

**Keywords:** Lung cancer, Macroautophagy

## Abstract

Y-box binding protein 1 (YBX1) is involved in the development of multiple types of tumors. However, the relationship between YBX1 and autophagy in non-small cell lung cancer (NSCLC) remains unclear. In this study, we analyzed the expression and clinical significance of YBX1 and markers of autophagy (LC3I/II) in NSCLC and examined their roles in regulating sensitivity to cisplatin in NSCLC. The retrospective analysis of patients with NSCLC indicated that YBX1 was positively correlated with autophagy. Increased levels of YBX1 or autophagy also observed in NSCLC cells compared with those in 16HBE cells. Compared to the controls, the knockdown of YBX1 expression suppressed autophagy, increased drug sensitivity and promoted apoptosis in response to cisplatin in NSCLC cells by targeting the p110β promoter and inhibiting p110β/Vps34/beclin1 signaling pathways. We also demonstrated in an in vivo study that the overexpressed YBX1 effectively increased NSCLC growth and progression and decreased the sensitivity to cisplatin by inducing autophagy in a xenograft tumor model, and these effects were concomitant with the increasing of p110β and beclin1 expression. Collectively, these results show that YBX1 plays an essential role in autophagy in NSCLC.

## Introduction

Lung cancer, a malignant lung tumor with uncontrolled cell growth in the lung tissue, is the main cause of cancer death worldwide^1,2^. Non-small cell lung cancer (NSCLC) accounts for approximately 85% of lung cancers^3^. Patients with lung cancer have short average survival time from the time of diagnosis. Because curative treatment is not usually feasible due to tumor dissemination, invasion, distant metastases and limited sensitivity to chemotherapeutic drugs^4^. Poor prognosis is closely related to drug insensitivity in NSCLC. Therefore, investigating mechanisms to increase the sensitivity to antitumor drugs in NSCLC has become urgent.

Autophagy plays an important role in cellular pathways, including growth, development, differentiation, aging, drug resistance and cell death^5,6^. Some reports suggest that this process can function as a tumor-suppression mechanism, while other data indicate that tumors may require autophagy to survive in nutrient-limited and low-oxygen conditions^7,8^. Several reports have shown that autophagy enhances the sensitivity to anti-tumor drugs in cancers. In contrast, autophagy also plays a key role in supporting cell survival during drug treatment^9,10^. These findings have suggested that manipulation of autophagy could have potential as a novel therapeutic strategy in cancer.

Y-box binding protein 1 (YBX1), a multifunctional protein that regulates transcription by binding to the Y-box (an inverted CCAAT box) at the promoter or enhancer of target genes, plays important pro-oncogenic roles in tumor invasion and metastasis^11–13^, drug resistance^14^, cell proliferation^15^ and DNA repair^16,17^. Recent studies have shown that YBX1 is associated with autophagy in intrahepatic cholestasis during pregnancy^18^. Nevertheless, the relationship and molecular mechanism between YBX1 and autophagy in NSCLC has not been elucidated.

In this study, we showed that the expression of the autophagy-associated protein LC3I/II was closely related to the expression of YBX1 in NSCLC tissue samples and cells, LC3I/II was controlled by YBX1, in accordance with our hypothesis. Simultaneously, the overexpression of YBX1 caused the upregulation of autophagy in vitro and in vivo. We discovered that the knockdown of YBX1 enhanced the antineoplastic effect of cisplatin by regulating autophagy. We also found that YBX1 promotes autophagy via the p110β/Vps34/beclin1 pathway, which suggested that the YBX1/p110β/Vps34/beclin1 axis is involved in cancer development and the progression of NSCLC.

## Materials and methods

### Cell lines and culture

Four NSCLC cell lines (A549, H1299, H460, and Hcc827) and one normal human bronchial epithelial cell line (16HBE) were purchased from the American Type Culture Collection (ATCC, Manassas). A549 and 16HBE were maintained in Dulbecco’s modified Eagle’s medium supplemented with 10% fetal bovine serum (FBS). H1299, H460, and Hcc827 cells were cultured in RPMI 1640 medium containing 10% fetal bovine serum (FBS). All the cells were maintained in a humidified atmosphere with 5% CO_2_ at 37 °C.

### Plasmid vector and small-interfering RNA

GeneCopoeia^TM^ supplied YBX1 overexpression plasmid (EX-Z2227-Lv201), p110β overexpression plasmid (EX-T0817-Lv206) and the control plasmid. GFP-LC3 plasmids (11546) were supplied by add gene. The shRNA expression vector for silencing YBX-1 and negative control vector were generated by our laboratory: (pGPU6/GFP/Neo-YBX1-homo-746 with the target sequence GGTTCCCACCTT ACTACAT; pGPU6/ GFP/Neo-YBX1-homo-326 with the target sequence AGAAG GTCATCGCAACGAA) and negative control vector (pGPU6/GFP/Neo-shNC) contain a selectable marker GFP. YBX1 siRNAs, beclin1 siRNAs, Bcl-2 siRNAs and their controls were supplied by Suzhou GenePharma. nonspecific siRNA (sense: 5′-UUCUCCGAACGUGUCACGUTT-3′, antisense:5′- ACGUGACACGUUCGGAGA ATT-3′). siRNAs for YBX1(sense: 5′-GGUUCCCACCUUACUACAU-3′, antisense: 5′- AGAAGGUCAUCGCAACGAA-3′; sense: 5′-UUCUCCGAACGUGUCACG UTT-3′, antisense: 5′-ACGUGACA CGUUCGGAGAATT-3′). siRNAs for p110β (sense: 5′-GCAACAGCUUUGCAUGUUATT-3′, antisense:5′-UAACAUGCAAA GCUGUUGCTT-3′). siRNAs for Bcl-2 (sense: 5′-GGGAGAUAGUGAUGAAGU ATT-3′, antisense:5′-UACUUCAUCACUAUCUCCCTT-3′. siRNAs for beclin1 (sense:5′-GGAGCCAUUUAUUGAAACUTT-3′, antisense:5′-AGUUUCAAUAA AUGGCUCCTT-3′). Invitrogen™ Lipofectamine™ 2000 Transfection Reagent (Thermo Fisher) was used to cell transfection.

### Western blot

Proteins from cell and tissue lysate were separated by 10–12% SDS-PAGE, transferred to polyvinylidene fluoride membranes, and immunoblotted with antibodies (1:1000) against YBX1 (ab12148,Abcam); LC3I/II(#12741,CST), cleaved-caspase-3 (#9961, CST), caspase-3(#9961, CST), caspase-9(#9961, CST), mTOR(#9862, CST), p-mTOR(Ser2448) (#9862, CST), p-p70s6k(Thr389) (#9862, CST), p70s6k (WL03 839,Wanleibio), AKT(#4060,CST), p-AKT (Ser473) (#4060,CST), GFP (#2555,CST); Bcl-2(12789-1-AP,Proteintech), β-actin(20536-1-AP,Proteintech), GAPDH(10494-1-AP, Proteintech), LaminB (12987-1-AP,Proteintech); p110β (sc-376 641, SANTA CRUZ), beclin1 (WL02508,Wanleibio). goat-anti-rabbit IgG conjugated to horseradish peroxidase (HRP) (HAF008, Proteintech); and goat-anti-mouse IgG conjugated to HRP (HAF007, Proteintech) which was used as the secondary antibody. Then immunoreactive protein bands were detected using ECL (Electrochemiluminescence) substrates.

### RT-PCR

Total RNA was extracted and reverse transcribed into cDNA according to the instructions of an RNAiso Plus kit and PrimeScript TM RT reagent Kit with gDNA Eraser (TaKaRa, China). The cDNA was amplified by PCR following the manufacturer’s protocol from TaKaRa Taq TM Hot Start Version (TaKaRa, China). The primers were as follows: p110β forward 5′-CCTTCGATAAGAGTCGAGGTGG-3′, reverse 5′-AACAGGTATGCATGGCCTCC-3′; YBX1 forward 5′-TGCAGCA GACCGTA ACCATT-3, reverse 5′-TGGATCGGCTGCTTTTGTC-3′; β-actin forward 5′-CCACCATGTACCCTGGCATT-3′, reverse 5′-ACTCCTGCTTGCTGATCCAC-3′; GAPDH forward 5′- GAGAAGGCTGGGGCTCATTT-3′, reverse 5′-AGTGAT GGCATGGACTGTGG-3′.

### Cell viability assay

We determined cell viability by MTT assay (Roche Diagnosis, Indianapolis, IN). Cells were added to a 96-well plate (3000 cells/well) and cultured overnight, then continuously cultured in a medium free of FBS. Six hours later, cells were transfected with siRNA or plasmid. After transfection for 6–8 h, cisplatin was added to the well. After 48 h of treatment, MTT was added to the cells and was incubated for 4 h. Then, the absorbance value at OD490 was measured.

### Caspase-3 activity assay

H1299 and A549 cells were treated with YBX1 sh-RNA or overexpression plasmids. After transfection for 6–8 h, cisplatin was added. After 48 h of treatment, cells were collected and centrifuged, adding 100 μL pyrolysis solution for 15 min, centrifuged at 4 °C for 15 min. Then reagents and samples were added one by one according to the designed protocol, the cells were incubated at 37 °C for 1 h. Then, the absorbance value at OD405 was measured.

### Apoptosis assay

The cells were grown in 6-well plates and then were transfected with YBX1-specific siRNA and negative control siRNA. After transfection for 6–8 h, the cells were treated with cisplatin for 48 h. Then, the cells were trypsinized without ethylenediaminetetraacetic acid (EDTA), washed twice with cold PBS and centrifuged. The cell pellet was re-suspended in 300 μL cold binding buffer, 3 μL Annexin V-FITC was added and mixed, and then 3 μL Propidium iodide was added and mixed. Detection of cell apoptosis was performed with FACS analysis with FITC-AV/PI staining.

### DNA-protein binding with a streptavidin-agarose pulldown assay

Binding of YBX1 to p110β core promoter probes was determined with a streptavidin-agarose pulldown assay. A biotin-labeled double-stranded probe corresponding to the p110β promoter sequence was synthesized. The binding assay was performed by mixing 400 μg of nuclear extract proteins, 4 μg of the biotinylated DNA probe and 40 μL of 4% streptavidin-conjugated agarose beads at room temperature for 1 h in a rotating shaker. Beads were pelleted by centrifugation to pull down the DNA-protein complex. After washing, the proteins in the complex were analyzed by immunoblotting using antibodies specific for YBX1.

### Transmission electron microscopy analysis

Standard transmission electron microscopy (TEM) was performed as previously described. Briefly, 48 h after YBX1 overexpression in A549 and Hcc827or silencing in H1299 and H460, the cells were fixed and embedded. Thin sections (90 nm) were examined at 80 kV with a JEOL 1200EX transmission electron microscope. Approximately 15 cells were counted.

### Immunofluorescence and confocal microscopy

Cells were cultured on glass slides located in a 6-well plate. After treatment for the desired time, fixed with 4% paraformaldehyde for 30 min, permeabilized with 0.2% Triton X-100 for 5 min and then blocked with blocking buffer (10% BSA in PBS) for 30 min, then they were incubated with primary antibody LC3I/II (#12741, CST,1:200) for 12 h, secondary fluorescent antibodies (ab150088, Abcam, 1:200) were added for 1 h. The cell nuclei were stained with DAPI for 5 min. Protein localization was analyzed using a confocal microscope (Leica) and processed using Image-Pro Plus 5.1 software (Media Cybernetics, Inc.)

### Acridine orange/ethidium bromide fluorescence staining(AO/EB)

H1299 and A549 cells were grown on chamber slides and treated with the indicated doses of cisplatin with or without melatonin. After 24 h, cells were washed with PBS to remove the detached cells and then fixed with 95% ethanol for 15 min. After slightly drying the cells, 5 µL AO/EB (50 µg/mL) was added and was gently pipetted to mix before imaging with a Leica DM 4000B microscope fitted with a digital camera.

### Patient characteristics and tissue preparation

NSCLC tissues from 100 patients (median age: 64 years; range 45 from 81 years) who underwent complete surgical resection of NSCLC were consecutively obtained at the First Affiliated Hospital of Dalian Medical University between January 2008 and December 2010. The 8th Edition International Union Against Cancer/American Joint Committee on Cancer TNM classification^19^ was applied to all enrolled patients. All treatment procedures followed the guidelines for the treatment of non-small cell lung cancer of the Chinese Society of Clinical Oncology (http://www.csco.org.cn/) and the National Comprehensive Cancer Network (https://nycancer.com/nccn/). Baseline data and clinicopathological information were collected from the medical record system (Dr. Fengzhou Li and Dr. Tao Guo). Postoperative follow-up was completed by outpatient follow-up and telephone interviews (Dr. Fengzhou Li and Dr. Shilei Zhao). Patients who received chemotherapy or radiotherapy prior to the operation were excluded. The patient follow-up was performed according to our previously reported process and ranged from 1 to 60 months after the primary operation (median follow-up time 43.5 months), and any patient that was lost to follow-up or died of causes other than lung cancer was recorded as censored at the time of the most recent follow-up. The study was approved by the Medical Ethical Committees of the First Affiliated Hospital of Dalian Medical University. All patients were informed of the study. All specimens were obtained from primary lesions, fixed with formalin, embedded with paraffin, and continuously sliced at a thickness of 4 μm.

### Immunohistochemistry staining

IHC staining of YBX1(ab12148,Abcam, 1:100), p110β(WL02849, Wanleibio, 1:100), beclin1 (WL02508, Wanleibio, 1:100) and LC3I/II (#12741, CST, 1:50) was performed according to the manufacturer’s instructions of the products used in our previous study^20,21^. A streptavidin–peroxidase staining kit was purchased from ZSGB BIO (Beijing, China). The paraffin-embedded tissues were dewaxed and rehydrated. The tissues were incubated with primary antibodies diluted to the recommended concentration overnight at 4 °C. Then, 3,3′-diaminobenzidine (DAB) staining was performed, and the results were observed under a microscope. The evaluation of expression was conducted by three professional pathologists separately. Any dispute was settled by the majority rule. The expression levels of the proteins were evaluated according to the methodologies indicated in previous reports.

### Animal study

Thirty male BALBc/nu mice (4–6 weeks old; 16–18 g weight) were obtained from Dalian Medical University and were maintained in an SPF Animal Center All animal maintenance and procedures were carried out in accordance with the National Institutes of Health Guide for the Care and Use of Laboratory Animals and were approved by the Animal Care and Ethics Committee of Dalian Medical University. To assess the effect of YBX1 on autophagy and the cisplatin response in vivo, nude mice were randomly divided into 4 groups (YBX1 Lacz group, *n* = 8; YBX1 Lacz with cisplatin injection group, *n* = 7; YBX1 OE group, *n* = 8; YBX1 OE with cisplatin injection group, *n* = 7) and were subcutaneously injected with 1 × 10^6^ cells (A549) near the axillary fossa. Two weeks later, when the tumor volume had reached approximately 20 mm^3^, cisplatin was dissolved in 0.9% NS (3 mg/kg) and was injected nto the tumor twice a week for 3 weeks. Tumor volume and body weights were measured twice a week. Tumor volume was calculated as V = 1/2 (width × length)2. Mice were humanely sacrificed by euthanasia after treatment.

### Statistical and data analysis

Each experiment was repeated 3 times under the same conditions. The results are shown as means ± standard deviation (SD) and were statistically analyzed using GraphPad Prism software version 5.01 (GraphPad Software, Inc., La Jolla, CA, USA). Student’s *t*-test was used to compare the values of the test and control samples, and a *P* value of less than 0.05 or 0.01 was considered to be statistically significant.

## Results

### YBX1 and autophagy-associated protein LC3I/II were co-highly expressed and positively correlated in patients with NSCLC

**Fig. 1:**
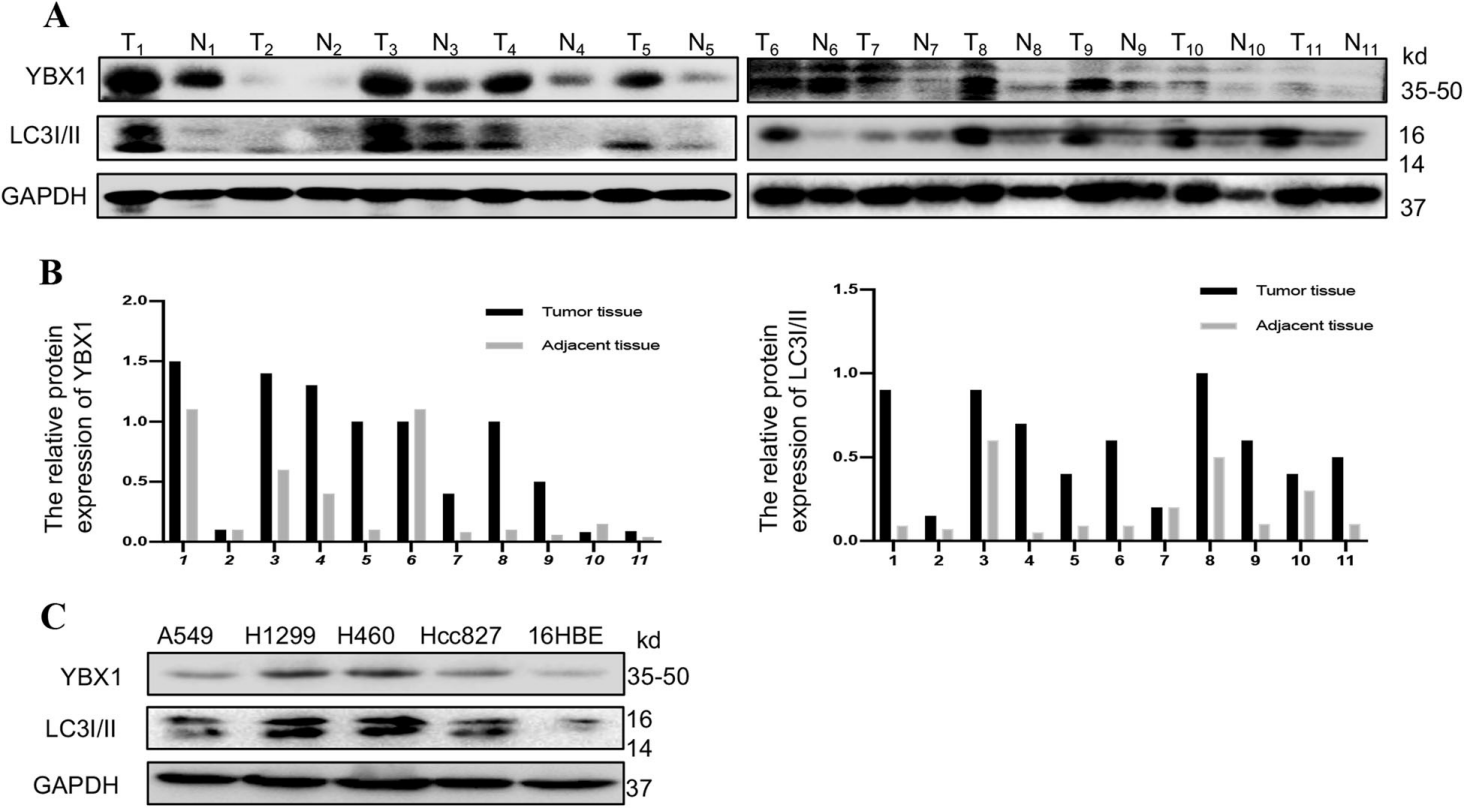
YBX1 and autophagy-associated protein LC3I/II were co-highly expressed and positively correlated in patients with NSCLC. **a** Protein samples were extracted from human NSCLC tissues and adjacent normal tissues, the expression of YBX1and LC3I/II was examined by western blotting (*n* = 11). Data represent the mean ± SD of three independent experiments. *P < 0.05 vs. control. **b** The distribution of NSCLC patients with YBX1 and LC3I/II expression in tumor tissues and adjacent tissues (*n* = 11). **c** YBX1 and LC3I/II proteins expression in a normal human bronchial epithelial cell line (16HBE) and various NSCLC cell lines (A549, H1299, H460, and HCC827) was analyzed by western blot.

We first examined by western immunoblotting the expression of YBX1 and LC3I/II in in the tumorous and paracancerous tissues from 16 NSCLC patients (Fig. 1a), in the human bronchial epithelial cell line 16HBE, and in 4 NSCLC cell lines (A549, H1299, H460, and HCC827) (Fig. 1b). The results showed that YBX1 and LC3I/II co-highly expressed in tumor cell lines or NSCLC tissues compared to their corresponding adjacent normal cells or normal tissues. These results suggest that YBX1was correlated with autophagy in NSCLC.

### YBX1 promotes autophagy in NSCLC cells

**Fig. 2:**
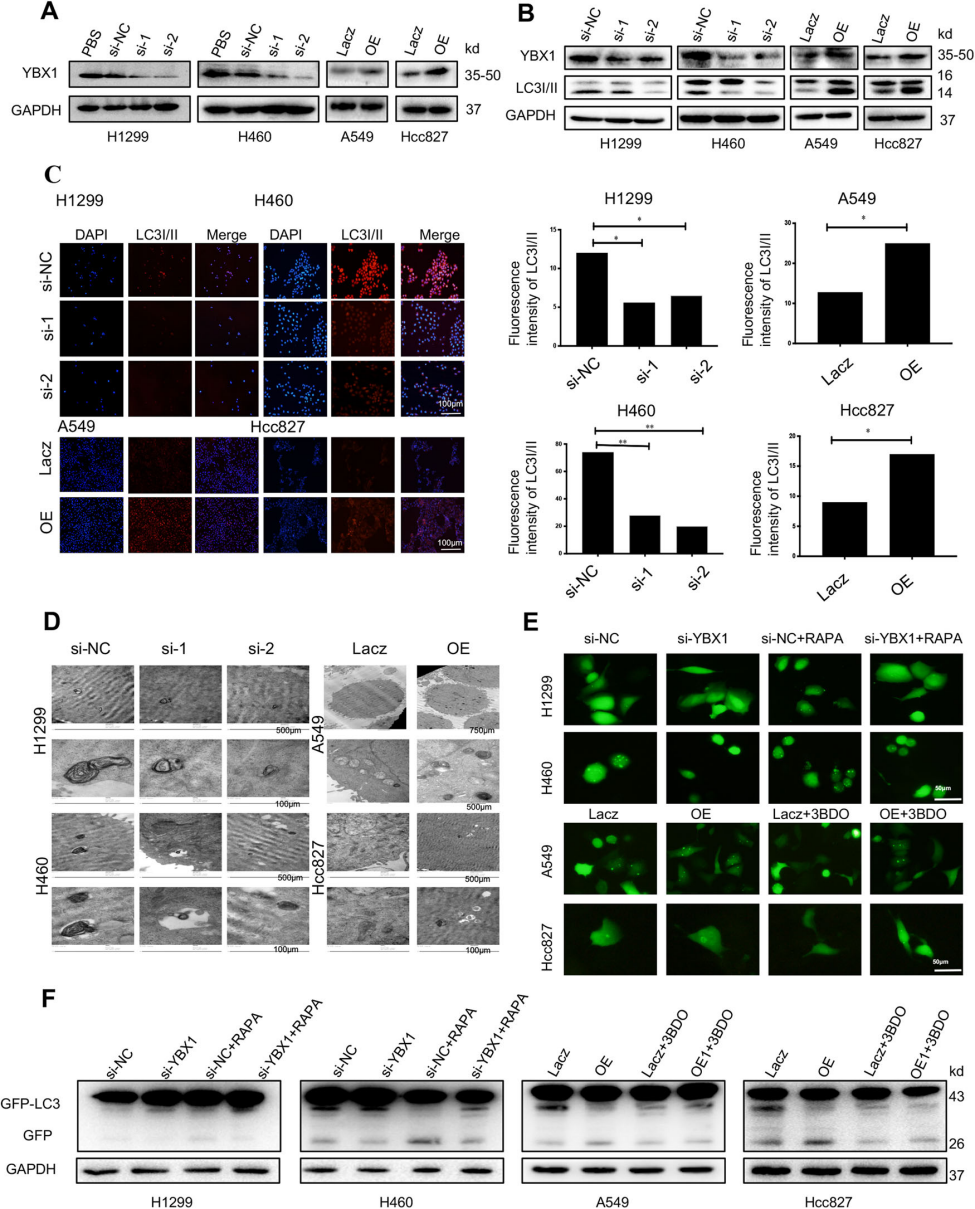
YBX1 promotes autophagy in NSCLC cells. H1299 and H460 cells were transfected with YBX1-specific siRNA, A549, and Hcc827 cells were transfected with YBX1-overexpression plasmids for 48 h. **a** YBX1 expression in H1299 and H460 cells were transfected with YBX1-specific siRNA or in A549 and Hcc827 cells were transfected with YBX1-overexpression plasmids was analyzed by western blot. **b** LC3I/II expression in H1299 and H460 cells were transfected with YBX1-specific siRNA or in A549 and Hcc827 cells were transfected with YBX1-overexpression plasmids was analyzed by western blot, (**c**) immunofluorescence, and (**d**) TEM-based ultrastructure analysis (autophagosomes). Data represent the mean ± SD of three independent experiments. *P < 0.05 and **P < 0.001 vs. control. **e** GFP-LC3 was co-transfected with YBX1 siRNA in H1299 and H460 cells, then treated with rapamycin at 471 nmol/L and 200 nmol/L, respectively, and co-transfected with YBX1-overexpressing plasmids in A549 and HCC827 cells, treated with 3BDO at 24 µmol/L and 120 µmol/L, respectively. The punctate GFP-LC3 was detected by immunofluorescence, and (**f**) the GFP-LC3 or GFP-LC3II conversation was detected by western blot. Data represent the mean ± SD of three experiments, *P < 0.05, **P < 0.01 vs. control.

To study the function of YBX1 on NSCLC autophagy levels. We transfected NSCLC cells with YBX1-targeting shRNA or overexpression plasmid, and after 48 h of treatment, the expression of YBX1 and LC3I/II were determined. The results showed that knockdown YBX1 effectively downregulated LC3I/II protein expression compared to the control, in contrast, the overexpression of YBX1 caused an increase of LC3I/II compared to the control (Fig. 2a–d). To further to determine whether YBX1 perturbs autophagosome formation of NSCLC cells, we detected GFP-LC3 dot formation, which is generally regarded as an autophagosome. It showed that the number of cell lines were transfect with GFP-LC3 after silencing YBX1 was significantly diminished compared to the control, and the cells treated with the autophagy inducer rapamycin exhibited typical GFP-LC3 dot formation. In contrast, the overexpression of YBX1 obviously enhanced these behaviors and the cells treated with autophagy inhibitor 3BDO demonstrated the restrain of GFP-LC3 dot formation (Fig. 2e, f). These results proved that YBX1 was involved in the regulation of autophagy in NSCLC cells.

### YBX1 targets p110β to induce autophagy with p110β/Vps34/beclin1 activation

**Fig. 3:**
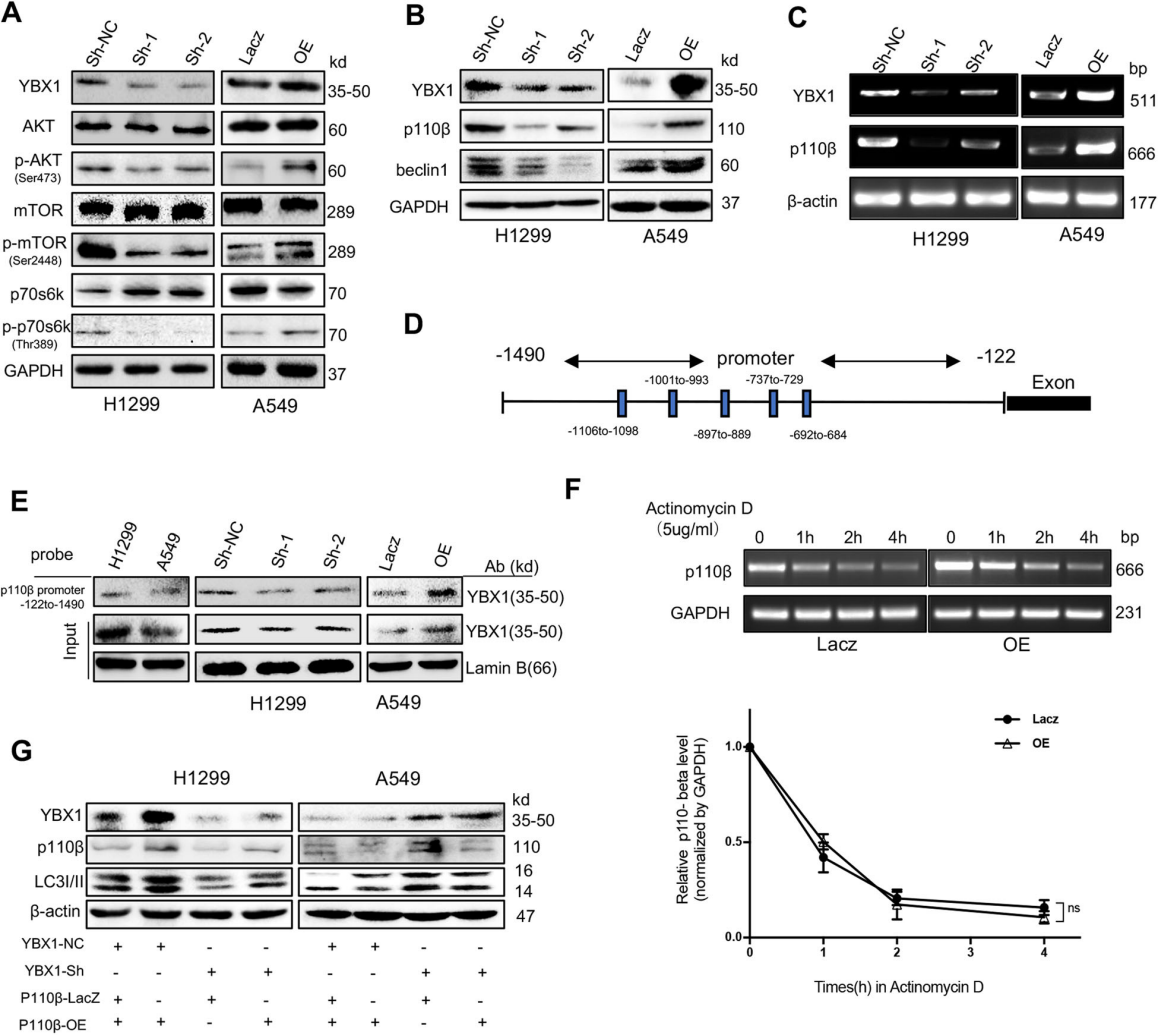
YBX1 targets p110β to induce autophagy with p110β/Vps34/beclin1 activation. H1299 transfected by YBX1-siRNA and A549 transfected by YBX1-overexpression plasmids, (**a**) The level of AKT, p-AKT(Ser473), mTOR, p-mTOR(Ser2448), p70s6k, p-p70s6k (Thr389), (**b**) p110β and beclin1 proteins in H1299 and A549 cells were analyzed by western blot, (**c**) The expression of the p110β mRNA was analyzed by RT-PCR. **d** YBX1 was predicted to bind the promoter of p110β and the data from the transcription binding site analysis(Jaspar), and the predicted binding sites of YBX1 on the promoter of p110β (score >7) were presented. **e** YBX1 proteins in the nuclear protein-p110β probe-streptavidin bead complexes were detected by western blot using an anti-YBX1 antibody in NSCLC cells. **f** A549 cells were transfected with YBX1-overexpression or vector plasmids, then added actinomycin D(5 μg/mL) at 0, 1, 2, 3, 4 h. The expression of the p110β mRNA was analyzed by RT-PCR. Data represent the mean ± SD of three experiments, *P < 0.05, **P < 0.01 vs. control. **g** H1299 cells were transfected with YBX1 sh-NC or sh-RNA overnight prior to transfection with the p110β control vector or overexpression plasmids. A549 cells were treated with the control vector or YBX1-overexpression plasmids overnight and then treated with p110β si-NC or siRNA for 48 h. The expression of YBX1, p110β, and LC3I/II was detected by Western blot.

Autophagy is regulated by a number of kinases, including PI3K/Akt/mTOR and p110 β/Vps34/beclin1 pathways, which negatively (the former)^22,23^ or positively (the latter)^24,25^ regulate autophagosome formation. To determine the effect of YBX1 on autophagy signaling, we transfected H1299 cell lines with YBX1 shRNA and A549 cell lines with YBX1 overexpressed plasmid, compared to the controls, YBX1 silencing led to suppress the phosphorylation of AKT, mTOR, and p70S6k. Inversely, overexpressed YBX1 increased them (Fig. 3a). About another pathway, compared to the controls, YBX1 knockdown led to the decreased expression of p110β and beclin1, overexpressed YBX1increased the expression of these proteins (Fig. 3b). These results suggest that the mTOR pathway, the negative regulation in autophagy, does not correspond to the influence of YBX1 on autophagy, however, p110β/Vps34/beclin1 pathway, the positive one was confirmed that YBX1 regulates autophagy by partly passed.

To identify the underlying molecular mechanisms by which YBX1 promoted autophagy in NSCLC, we examined the effects of YBX1 on the p110β/Vps34/beclin1 pathway, given its key role in mediating autophagy. We found YBX1 knockdown was accompanied by a marked reduction ofp110β at the level of mRNA compared to those in the controls. In contrast, the overexpression of YBX1 increased the expression compared to those in the controls (Fig. 3c). Because YBX1 is a known transcriptional activator, we set out to mechanistically interrogate its function in the regulation of p110β expression, we used the analysis of transcription factor prediction software, found that YBX1 could be combined to the promoter of p110β (Fig. 3d). We designed a biotinylated-p110β promoter probe, pulldown assay showed that YBX1 bind to the p110β promoter probe, corresponding to the sequence −1490 to −122, was observed. Compared to the controls, YBX1 silencing inhibited the level of YBX1 binding to the p110β promoter, and YBX1 overexpression enhanced the binding (Fig. 3e).

To further to confirm whether YBX1 enhances the expression of p110β mRNA or proteins at the transcriptional level instead of causing a reduction in protein degradation, we measured the decay curve of p110β mRNA in A549 cells after overexpressed YBX1 and treated with actinomycin D to inhibit the activity of RNA polymerase II at different time points. It showed that the attenuation curve of p110β mRNA had no significant in YBX1 overexpressed group than control, and increased the expression of YBX1 protein could not significant decay the degradation of p110β mRNA (Fig. 3f). It showed that YBX1 promotes autophagy through enhancing p110β transcription.

To determine whether autophagy was influenced by YBX1-induced p110β expression, we silenced YBX1 and overexpressed p110β in H1299 cells or overexpressed YBX1 and silenced p110β in A549 cells to rescue the YBX1-mediated p110β protein expression (Fig. 3g). These results showed that YBX1 activates the promoter of p110β (−1490 to −122) to induce autophagy in NSCLC cells.

### YBX1 decreased the cisplatin-mediated inhibition of cell proliferation and enhancement of cell apoptosis by induce autophagy in NSCLC

**Fig. 4:**
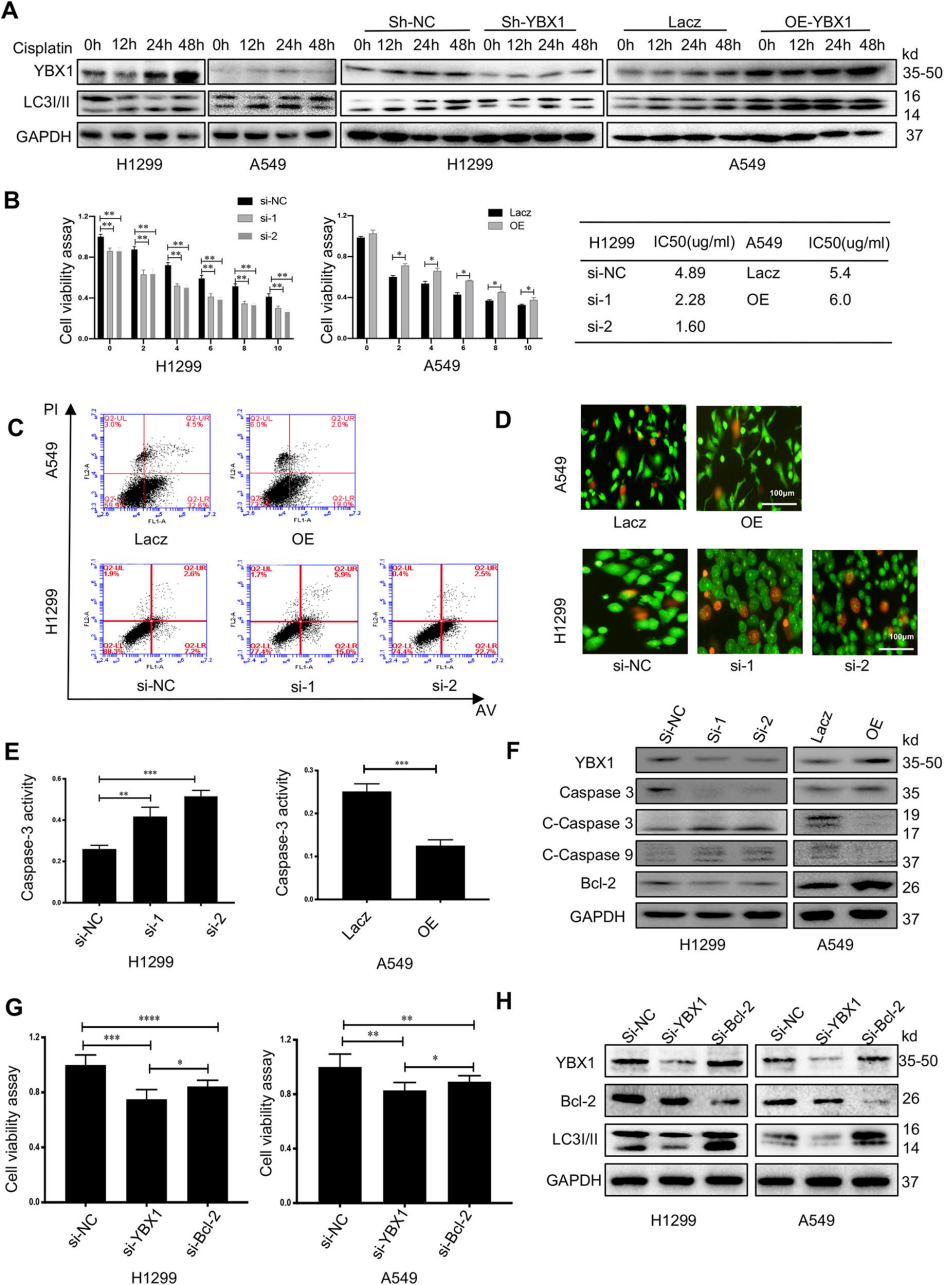
YBX1 decreased the cisplatin-mediated inhibition of cell proliferation and enhancement of cell apoptosis by induce autophagy in NSCLC. **a** H1299 and A549 cells with or without YBX-siRNA or overexpressing plasmid were treated with cisplatin at 0, 12, 24, and 48 h, and the proteins levels of YBX1 and LC3I/II a were detected by western blot. **b** The NSCLC cells were treated with various concentrations of cisplatin (0, 2, 4, 6, 8, or 10 μg/mL) after transfection with YBX1-siRNA in H1299 cells or with a YBX1-overexpression plasmid in A549 cells for 48 h. Cell viability was analyzed by MTT. Data represent the mean ± SD of three experiments, *P < 0.05, **P < 0.01 vs. control. **c** FACS analysis, (**d**) AO/EB, (**e**) Caspase-3 activity and (**f**) western blot (the levels of proteinYBX1, caspase-3, cleaved-caspase-3,9, and Bcl-2) were performed after YBX1-siRNA transfected in H1299 cells and cisplatin treated with 6.19 μg/mL or YBX1-overexpression plasmid transfected in A549 cells and cisplatin treated with 6.1 μg/mL. Data represent the mean ± SD of three experiments, *P < 0.05, **P < 0.01 vs. control. **g** Cell viability assay was performed after NSCLC cells transfected with YBX1 siRNA or Bcl-2. Results are expressed as mean ± SD of three independent experiments, *P < 0.05, **P < 0.01 vs. control. **h** The expression of YBX1, Bcl-2, and LC3I/II was analyzed by western blot after NSCLC cells transfected with YBX1 siRNA or Bcl-2. Results are expressed as mean ± SD of three independent experiments, *P < 0.05, **P < 0.01 vs. control.

YBX1 protein expresses higher in cisplatin-resistant cell lines than that in sensitive ones^26^. To investigate whether YBX1 affects the cell proliferation inhibition mediated by cisplatin, we treated the NSCLC cell lines with cisplatin in the indicated does at different times. We found that YBX1, LC3I/II conversion increased in a time-dependent manner compared to that in the controls, Whether YBX1 knocked-out or overexpressed, the changes of LC3I/II conversion were in a time dependent in NSCLC cells (Fig. 4a). Then, we treated various concentrations of cisplatin (0, 2, 4, 6, 8, or 10 μg/mL) after YBX1 knockdown in H1299 or overexpressed in A549 cells, the cell viability was determined. YBX1 markedly decreased the cisplatin-mediated suppression on cell viability compared to that in the controls (Fig. 4b). To assess the effect of combined YBX1 modulation and cisplatin on apoptosis in NSCLC cells, we detected by flow cytometry, AO/EB and western blotting assay. Our results showed that YBX1 silencing increased apoptosis (Fig. 4c, d), activated Caspase-3, downregulated the expression of Bcl-2 and Caspase 3, upregulated the expression of Cleaved-Caspase 3/9 compared to the controls (Fig. 4e). In contrast, the overexpression of YBX1 obviously enhanced these behaviors in A549 cells compared to those in the controls. These results demonstrated that knockdown of YBX1 resulted in enhanced cisplatin anti-proliferative effects in NSCLC.

To determine the potential molecular mechanism governing the ability of YBX1 to inhibit the sensitivity to cisplatin of NSCLC cells, we added the cisplatin in NSCLC cells after transfect YBX1 siRNA and Bcl-2 siRNA. Our results showed that silencing YBX1 and Bcl-2 increased the sensitivity to cisplatin compared to the control, but knocking down YBX1 is better (Fig. 4f).

Previous studies have shown that there is a close relationship between YBX1 and BCL-2^27^. Bcl-2 as an anti-apoptotic protein inhibits beclin1-dependent autophagy^28^. To further to confirm the signaling of YBX1 on autophagy, we analyzed the effect of YBX1 and Bcl-2 in H1299 and A549 cells. We found that YBX1 knockdown accompanied with Bcl-2 and LC3I/II decreased, but Bcl-2 knockdown accompanied by the increase of LC3I/II (Fig. 4g). We had showed that silencing YBX1 decreases autophagy in NSCLC cells, these results suggested that autophagy may be involved in YBX1-mediated sensitivity to cisplatin in NSCLC cells.

### YBX1 promotes autophagy and mediates sensitivity to cisplatin in a mouse model

**Fig. 5:**
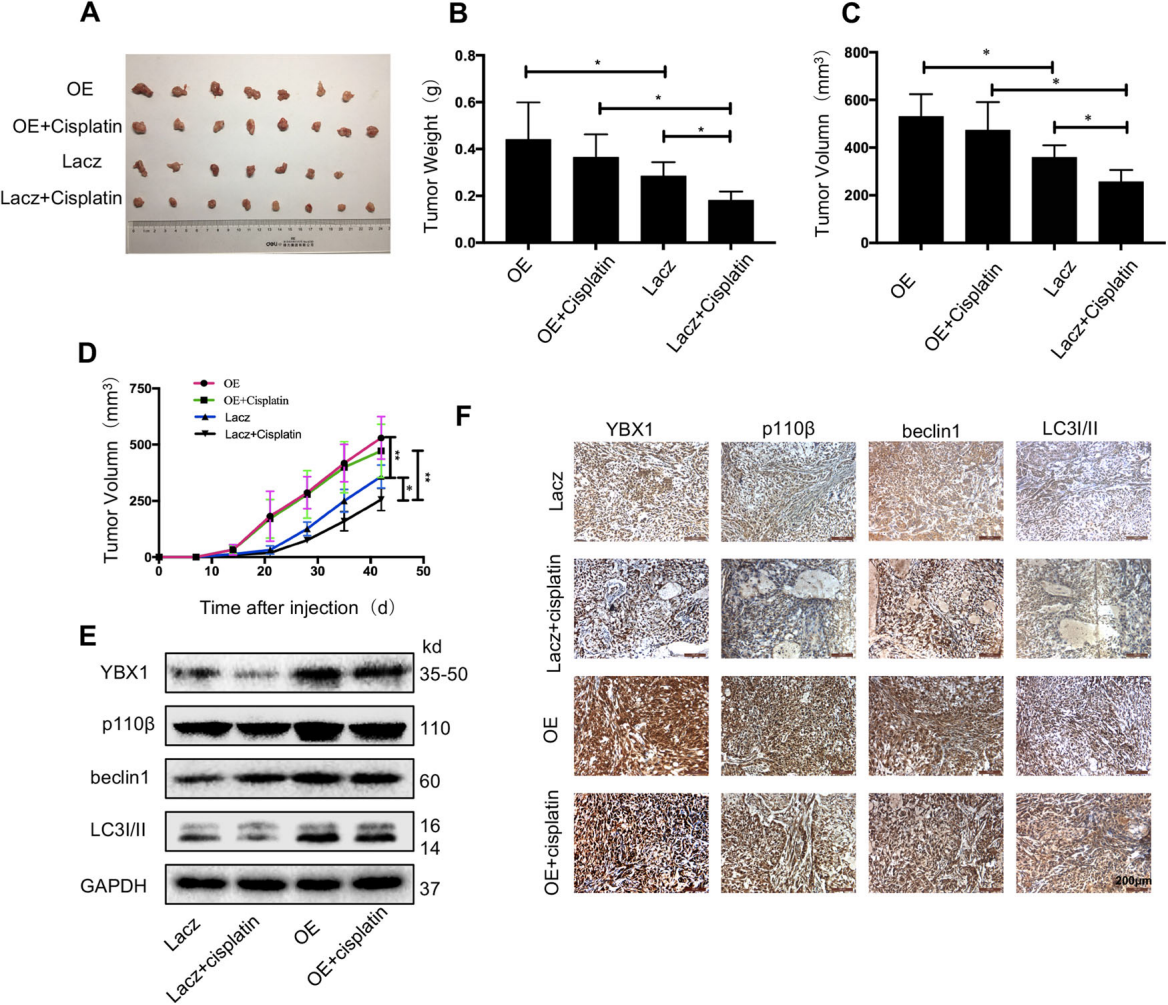
YBX1 promotes autophagy and mediates sensitivity to cisplatin in a mouse model. YBX1-overexpressing A549 cells and the corresponding control cell lines were injected into the left flank of nude mice to evaluate the effects of different treatments on tumor growth and autophagy. The four treatment groups were used: (1) Lacz + NS (*n* = 7); (2) Lacz+cisplatin (*n* = 8); (3) YBX1 OE + NS (*n* = 7); and (4) YBX1 OE + cisplatin (*n* = 8). **a** Tumors from mice. (**b**) Tumor weights. **c** Tumor volume of nude mice from each group was measured and calculated as volume = (width^2 ^× length)/2. **d** Tumor growth kinetics. **e** Western blot analysis of YBX1, p110β, beclin1, and LC3I/II from tumor tissues of mice. **f** Immunohistochemical analysis of YBX1, p110β, beclin1, and LC3I/II from tumor xenografts. Data are expressed as mean ± SD, *P < 0.05, **P < 0.01.

To further examine the effects of YBX1 on tumor growth, drug sensitivity and autophagy via p110β signaling in vivo, we used nude mice bearing NSCLC xenografts. The A549 and control cells bearing a stable overexpress of YBX1 were injected subcutaneously into the underarms of nude mice. Tumors derived from A549 cells with the stable overexpression of YBX1 were bigger and heavier than controls (Fig. 5a, d). To further verify the effect of YBX1 on sensitivity to cisplatin of NSCLC cells, we used cisplatin on nude mice on the seventh day after injection of tumor cells. We found that the YBX1-overexpressed group was less sensitive than the control group in responding to radiotherapy. In addition, we examined the effect of YBX1 overexpress on the expression of tumor-associated protein levels in xenografts by western blotting (Fig. 5e) and IHC analysis (Fig. 5f), and the results showed that YBX1 overexpression considerably increased the p110β, beclin1, LC3I/II proteins expression in the YBX1 overexpression groups compared to the control groups. These data further demonstrate that the YBX1 mediates NSCLC growth, drug sensitivity and autophagy by activating p110β signaling in animals.

### YBX1 is positively correlated with LC3I/II expression in clinical tissue sample

**Fig. 6:**
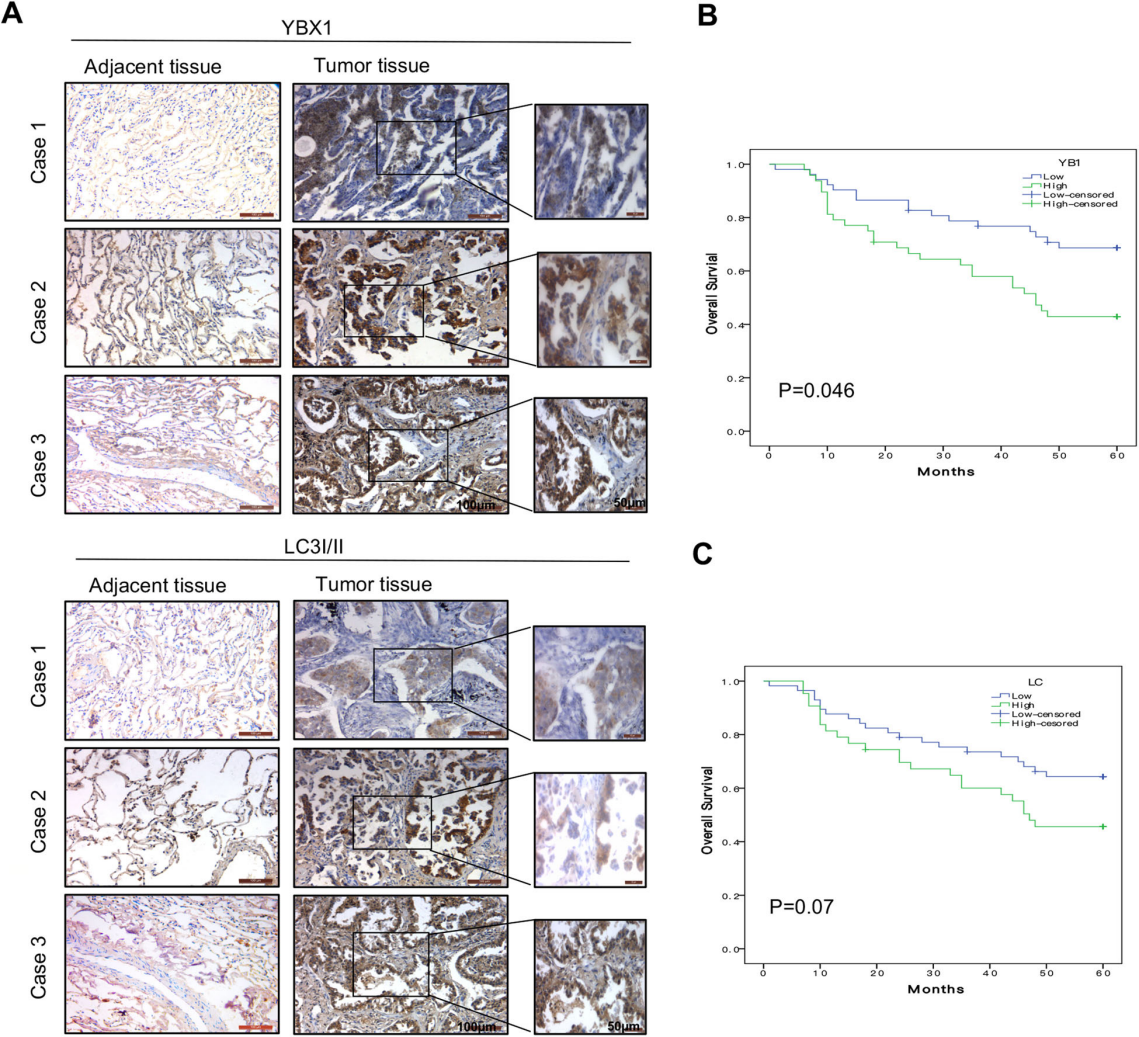
YBX1 is positively correlated with LC3I/II expression in clinical tissue samples. **a** The expression of YBX1 and LC3I/II protein from NSCLC tissue and and their adjacent lung tissues was analyzed by immunostaining analysis. **b** Kaplan–Meier analysis of overall survival for NSCLC patients with different expression levels of YBX1 by log-rank test. **c** Kaplan-Meier analysis of overall survival for NSCLC patients with different expression levels of LC3I/II by log-rank test.

To further confirm the involvement of YBX1 in autophagy-mediated NSCLC survival, we examined the expression of YBX1 and LC3I/II in clinical lung tumor tissue samples and analyzed the relationship of these proteins with the prognosis of patients with 100 NSCLC using IHC assay (Fig. 6a), and 48 (48%) patients showed high expression of YBX1, 43 (43%) patients showed high expression of LC3I/II. YBX1 expression was significantly correlated with smoking and the clinical TNM stage, and LC3I/II expression was significantly correlated with just the clinical TNM stage (Table 1). Patients with low YBX1 expression had significantly higher survival rates compared with that of patients with high YBX1 expression; however, the low expression of LC3I/II in the patients had no effect on the survival rates (Fig. 6b). In addition, a positive correlation between YBX1 and LC3I/II proteins expression was observed (Table 2). These results indicated that YBX1 was potentially synergistic with LC3I/II in the prediction of NSCLC and its expression in patients with NSCLC.

**Table 1:** Relations between the level of LC3I/II or YBX1 expression and clinicopathologic characteristics in 100 NSCLC.

Clinical factor	YBX1	Expression	*P* value	LC3I/II	Expression	*P* value
	High (%)	Low (%)		High (%)	Low (%)	
Over all	48	52		43	57	
Gender						
Male	23 (47.9)	24 (46.2)	0.156	18 (41.9)	29 (50.9)	0.422
Female	25 (52.1)	28 (53.8)		25 (58.1)	28 (49.1)	
Age						
<64	22 (45.8)	27 (51.9)	0.556	19 (44.2)	30 (52.6)	0.426
≥64	26 (54.2)	25 (48.1)		24 (55.8)	27 (47.4)	
Smoke						
Yes	29 (60.4)	20 (38.5)	0.045*	21 (48.8)	28 (49.1)	1.000
No	32 (61.5)	19 (39.6)		22 (51.2)	29 (50.9)	
T						
T1 40(40)	16 (33.3)	24 (46.2)	0.000*	6 (14)	34 (59)	0.000*
T2 37(37)	17 (35.4)	20 (38.4)		23 (53.5)	15 (24.6)	
T3 23(23)	15 (31.3)	8 (15.4)		14 (32.6)	9 (15.8)	
N						
N0 38(38)	19 (39.6)	19 (39.6)	0.000*	15 (34.9)	35 (61.4)	0.000*
N1 23(23)	15 (31.3)	8 (15.4)		10 (23.3)	13 (22.8)	
N2 27(27)	14 (29.1)	13 (25)		18 (41.9)	9 (15.8)	
TNM						
I 46(46)	18 (37.5)	28 (53.8)	0.000*	12 (27.9)	34 (59.6)	0.000*
II 20(20)	9 (18.8)	11 (21.2)		9 (20.9)	11 (19.3)	
III34(34)	21 (43.7)	13 (25)		22 (51.2)	12 (21.1)	

*P < 0.05.

**Table 2:** The positive correlation between YBX1 and LC3I/II protein expression in NSCLC.

YBX1 expression	LC3I/II	expression	*P* value
	High	Low	
High	26	22	0.043
Low	17	35	

*P < 0.05.

### The sensitivity to cisplatin was modulated by autophagy in NSCLC

As previous study showed, cisplatin induced autophagy in NSCLC cells, and we also found both mTOR and p110β/Vps34/beclin1 pathways are involved in cisplatin-induced autophagy of NSCLC cell lines (Fig. S1A). To further to elucidate the relationship between autophagy and cisplatin in NSCLC. we used the autophagy inducer rapamycin and the autophagy inhibitor 3BDO because these two compounds target mTOR1 to induce or inhibit autophagy (Fig. S1B). We also silenced beclin1 to inhibit autophagy in NSCLC cells (Fig. S1C). The results showed that rapamycin markedly decreased the cisplatin-mediated suppression of cell viability and enhancement of apoptosis. In contrast, 3BDO enhanced the suppression of cell viability and enhancement of apoptosis. We also found beclin1 knockdown enhanced cisplatin-mediated suppression of cell viability and enhancement of apoptosis compared to the control (Fig. S1D-E). These data indicate that autophagy may act as a survival mechanism in cells treated with cisplatin, and the attenuation of the autophagy enhances the response to cisplatin therapy in NSCLC cells.

## Discussion

Our study proved that both NSCLC cells and tissue specimens harbored high expression of YBX1 and LC3I/II. YBX1 overexpression remarkably promoted autophagy in vitro and in vivo. YBX1 decreased the sensitivity to cisplatin by partly inducing autophagy not just by increasing the expression of Bcl-2. Further integrated analyses showed that p110β is key effector that is regulated by YBX1 to mediate autophagy. These analyses illustrated the pivotal role of p110β/Vps34/beclin1 signaling in autophagy and the indispensable relationship of p110β in the YBX1-mediated transcriptional regulation of p110β/Vps34/beclin1. We also explored and confirmed that the sensitivity of NSCLC to cisplatin was regulated by YBX1 and showed that the high expression of YBX1 was a potential predictor of poor prognosis for patients with NSCLC. Moreover, we also demonstrated that the sensitivity to cisplatin was modulated was by autophagy. To the best of our knowledge, the autophagy-promoting role of YBX1 in NSCLC and the mechanistic insights into such function were not reported previously.

Some studies have shown that mTOR signaling functions as a classic negative pathway in the regulation of autophagy, and p110β/Vps34/beclin1 signaling is a newly discovered pathway that positively regulates autophagy^29,30^. Both mTOR and p110β signaling are positively controlled by YBX1^31,32^, and play crucial roles in the development of NSCLC. At the same time, the autophagy of NSCLC was regulated by YBX1, and a previous study has shown mTOR signaling regulates autophagy negatively, p110β/Vps34/beclin1 is positively. Thus, YBX1-mediated autophagy is possibly driven by the p110β/Vps34/beclin1 pathway and not by the mTOR pathway.

Next, we furthered proved that overexpression of YBX1 reduced the sensitivity of NSCLC to cisplatin in vitro and in vivo. Similarly, these effects were reversed with YBX1 knockdown. We found that YBX1 decreases the sensitivity by inducing autophagy not only by inhibiting Bcl-2. In addition, cisplatin increased LC3I/II conversion and YBX1 protein expression in a time-dependent manner and cisplatin induced autophagy by passing mTOR and p110β/Vps34/beclin1 signaling. We also found that the sensitivity to cisplatin was mediated by autophagy, showing that YBX1 is the key mediator of autophagy and thus in maintaining sensitivity to cisplatin. Most likely, the upregulation of autophagy that is mediated by YBX1 promotes cell proliferation, induces drug resistance, and antagonizes cell apoptosis in NSCLC.

Of note, further investigation showed the indispensable synergy of p110β in the YBX1-mediated transcriptional regulation of autophagy. Beyond that, the precise domain bound by YBX1 within p110β was clarified with a pulldown assay. YBX1 overexpression or silencing upregulated or downregulated its binding at the p110β promoter, suggesting that YBX1 directly binds to the p110β promoter. Moreover, consistent with this hypothesis, p110β knockdown reversed the YBX1 overexpression-induced elevation of LC3I/II, and vice versa, fully supporting the effect of p110β in YBX1-mediated autophagy effects in NSCLC. The precise role of YBX1 in mediating p110β function is still unclear and deserves to be better investigated in future studies.

The clinical application of cisplatin agents confers an overall survival advantage for patients with NSCLC harboring mutant YBX1^33,34^. However, the cessation of therapeutic effectiveness gradually develops because of acquired resistance to these agents. Several mechanisms that are responsible for acquired resistance have been described, including increasing the level of autophagy^10^. Given that the regulation of autophagy is mediated by YBX1 and cisplatin, our studies provide a hypothesis and evidence for the functional role of YBX1 expression in modulating acquired resistance to cisplatin therapies. We proved that overexpression of YBX1 reduced the sensitivity of NSCLC to cisplatin in vitro and in vivo. Similarly, these effects were reversed with YBX1 knockdown. We found that YBX1 decreases the sensitivity by inducing autophagy not only by inhibiting Bcl-2, showing that YBX1 is the key mediator of autophagy and thus in maintaining sensitivity to cisplatin. Most likely, YBX1 expression analysis could be performed during cisplatin therapy, especially upon the development of acquired resistance for this treatment.

In addition to YBX1, autophagy also regulated sensitivity to chemotherapeutics^35,36^. In our study, cisplatin increased LC3I/II conversion and YBX1 protein expression in a time-dependent manner and cisplatin induced autophagy by passing mTOR and p110β/Vps34/beclin1 signaling. We also use of an autophagy inducer (RAPA), inhibitor (3BDO) or beclin1 knockdown affects the level of apoptosis in NSCLC cells, and found that inhibiting autophagy can increase the sensitivity to cisplatin in NSCLC. They proved that the sensitivity to cisplatin is mediated by autophagy as well.

The clinical significance of YBX1 and autophagy in NSCLC was also shown. Among the 100 NSCLC patient samples, high YBX1 expression was observed in 48% of patient samples, and high LC3I/II expression was observed in 43% of the patient samples; both YBX1 and LC3I/II were statistically associated with the clinical TNM stage. Moreover, patients with high YBX1 expression had a significantly shorter overall survival time; however, LC3I/II expression had no effect on overall survival time, suggesting that YBX1 could potentially serve as a diagnostic marker and an independent prognostic predictor for NSCLC progression. In addition, the YBX1 and LC3I/II protein conversion from NSCLC tissues and cells were positively correlated.

In conclusion, our research showed that showed the sensitivity to cisplatin therapy is regulated by YBX1-mediated autophagy in NSCLC, we have also delineated the underlying molecular mechanisms: YBX1 elevates p110β/Vps34/beclin1 expression by directly binding to the p110β promoter, acting as a transcriptional activator. All of our evidence suggests that the combination of YBX1 and autophagy modulators could be an effective strategy in NSCLC treatment. In our study, we used H1299 cell line which is p53 deficient^37^. Previous study reports that p53 was involved in regulating autophagy^38^. So, we can not rule out the possibility of YBX1 regulating autophagy by interacting with p53. However, the study of YBX1 mediates p110β-associated pathway to regulate autophagy in NSCLC is still reliable. At the same time, we will further study whether the deletion of p53 gene has an effect on autophagy of NSCLC in the future.

## Supplementary information

Figure.S1

Figure legends for Figure S1
