# Supplementary figures and images for: YBX1 mediates autophagy by targeting p110β and decreasing the sensitivity to cisplatin in NSCLC
Source: Cell Death Dis. 2020 Jun 19;11(6):476. doi: 10.1038/s41419-020-2555-4 (PMC7305216; doi:10.1038/s41419-020-2555-4)

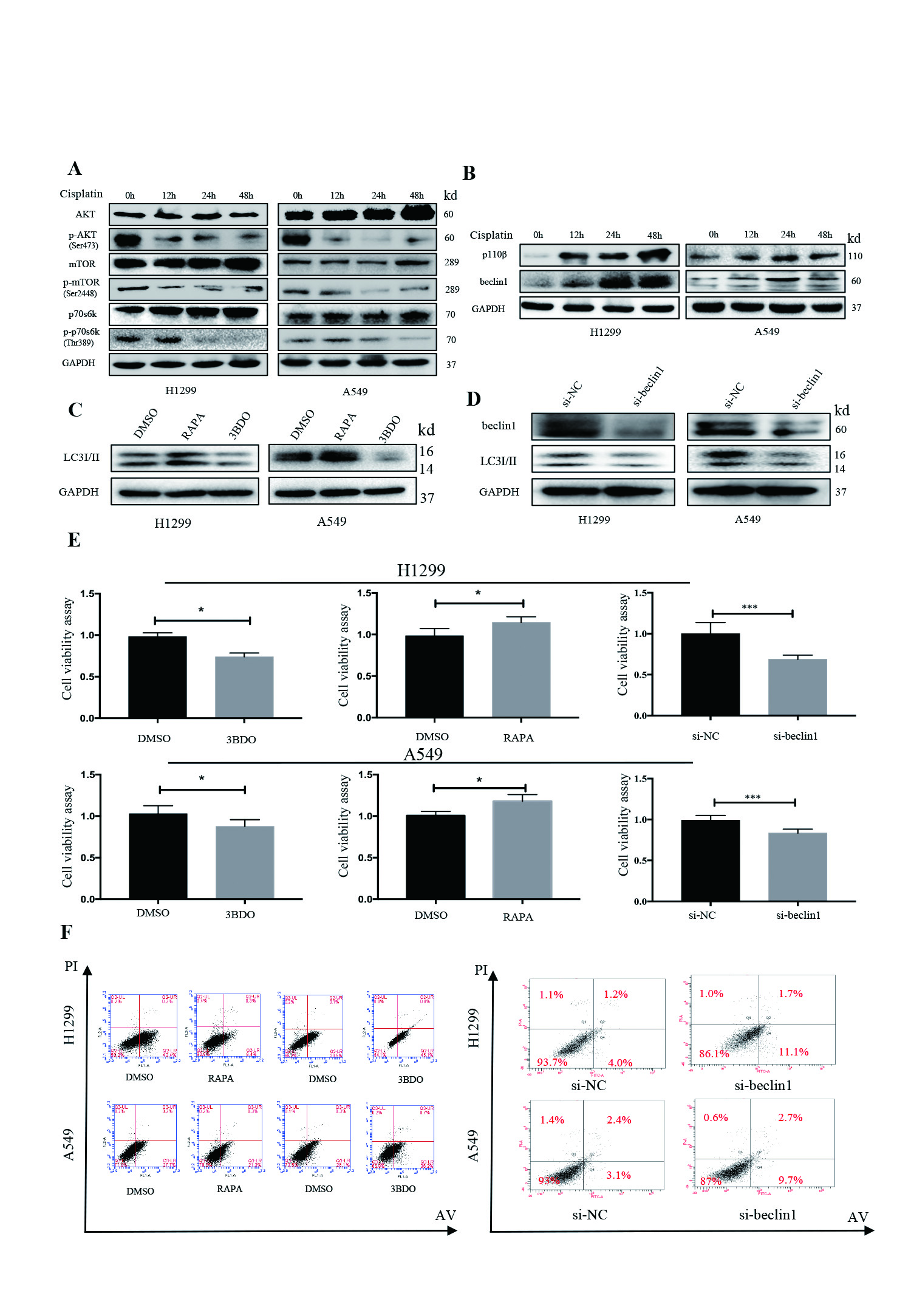

Supplement: Supplementary file 1 — Figure.S1 [file 41419_2020_2555_MOESM1_ESM.jpg]
