# Supplementary material for: YBX1 mediates autophagy by targeting p110β and decreasing the sensitivity to cisplatin in NSCLC
Source: Cell Death Dis. 2020 Jun 19;11(6):476. doi: 10.1038/s41419-020-2555-4 (PMC7305216; doi:10.1038/s41419-020-2555-4)
Supplement: Supplementary file 2 — Figure legends for Figure S1 [file 41419_2020_2555_MOESM2_ESM.docx]

**Fig. S1 The sensitivity to cisplatin was modulated by autophagy in NSCLC.** (A)The levels of AKT, p-AKT(Ser473), mTOR, p-mTOR(Ser2448), p70s6k, p-p70s6k (Thr389) and (B) The levels of p110β, beclin1 in H1299 and A549 cells were analyzed by western blot after treated with cisplatin at indicated does for 0, 12, 24, 48 h. (C) H1299 and A549 cells were treated with rapamycin (an autophagy inducer that inhibitors mTOR phosphorylation; H1299 471 nmol/L, A549 208 nmol/L) and 3BDO (an autophagy inhibitor that enhances mTOR phosphorylation; H1299 188 µmol/L, A549 240 µmol/L) for 4-6h, then cisplatin were added for 48h, the level of LC3I/II protein was detected by western blot. (D) H1299 and A549 cells transfected beclin1-siRNA, and cisplatin were added for 48h, the level of beclin-1 and LC3I/II proteins was detected by western blot. (E) Cell viability was analyzed by MTT and (F) FACS analysis was performed after H1299 and A549 cells induced or inhibited autophagy. Data are represented as mean ± SD of three experiments, *P < 0.05.
